# Center of pressure and ground reaction forces in Labrador and Golden Retrievers with and without hip dysplasia at 4, 8, and 12 months of age

**DOI:** 10.3389/fvets.2022.1087693

**Published:** 2022-12-22

**Authors:** Yvonne Virag, Michaela Gumpenberger, Alexander Tichy, Christiane Lutonsky, Christian Peham, Barbara Bockstahler

**Affiliations:** ^1^Section of Physical Therapy, Small Animal Surgery, Department of Companion Animals and Horses, University Clinic for Small Animals, University of Veterinary Medicine, Vienna, Austria; ^2^Diagnostic Imaging, Department of Companion Animals and Horses, University of Veterinary Medicine, Vienna, Austria; ^3^Department of Biomedical Sciences, Bioinformatics and Biostatistics Platform, University of Veterinary Medicine Vienna, Vienna, Austria; ^4^Department of Companion Animals and Horses, Movement Science Group, University Clinic for Horses, University of Veterinary Medicine, Vienna, Austria

**Keywords:** center of pressure, ground reaction forces, canine hip dysplasia, Labrador Retriever, Golden Retriever

## Abstract

Canine hip dysplasia (CHD) is a common orthopedic disease. Owing to the importance of CHD in affected dogs, both clinically and for their use in breeding or work, increasing attention is being given to early diagnosis. Therefore, early clinical and radiological examination of young animals is increasingly in demand, whereas common CHD screening according to the Fédération Cynologique Internationale (FCI) is usually performed at the age of 12 months or even older in Europe. One way to objectively evaluate gait pattern is to measure the ground reaction forces (GRFs) and center of pressure (COP). In this study, we used a pressure plate to evaluate the GRF and COP parameters for 32 Labrador Retrievers and 17 Golden Retrievers at 4, 8, and 12 months of age. The dogs also underwent radiological examination of the hip joints following the FCI rules at the age of at least 12 months, which were grouped as sound (FCI grade A or B) and diseased (FCI grade C or worse). The results revealed significantly higher COP values in both breeds in the diseased limb groups at any measurement point during walking, with the most pronounced results obtained at 8 months of age. Furthermore, COP values during walking were significantly higher at 4 months than at 8 and 12 months in both the sound and diseased limb groups, indicating an increased stability of the gait pattern. Except for COP-Speed, the values of all COP parameters were higher during walking than during trotting at 4 months of age (i.e., COP-Speed was higher when trotting), indicating that the 4-beat gait in walk is more difficult to control for puppies than the 2-beat gait in trot. Overall, our results support the early evaluation of CHD in growing animals using non-invasive methods.

## 1. Introduction

Canine hip dysplasia (CHD) is one of the most common orthopedic diseases in young dogs without a traumatic background, especially in large and predisposed breeds. This condition leads to significant orthopedic effects and pain in aging animals through the development of osteoarthritis (OA) (1, 2).

According to the rules of the Fédération Cynologique Internationale (FCI), radiographic examination for CHD screening should be performed at the age of 12 months (or even 18 months for large-breed dogs). However, owing to the importance of CHD in affected dogs, both clinically and for their use in breeding or work, increasing attention is being given to early diagnosis (3). Clinical and radiological examinations of young animals, even before official radiographic FCI hip scoring, are increasingly in demand. Clinical evaluation methods include the Ortolani, Bardens, and Barlow tests, (3), whereas the Penn Hip method is preferred for radiographs, in which a distraction index (DI) is used to determine hip joint laxity (4). In addition, other radiological parameters, such as dorsal acetabular rim slope and center edge angle, and qualitative parameters, such as sclerosis of the cranial acetabular rim, location of the center of the femoral head, grading of the degenerative joint disease, and grading of the dorsal acetabular rim, are also used (5). A total score that includes different parameters can be used as an early detection method to distinguish dogs that are later classified as CHD-free (according to the FCI guidelines) and dogs with a transitional hip (FCI-B) (6). Furthermore, the combination of the Norberg angle, distraction recording, and laxity index presents a reliable prediction of whether a dog will develop an FCI-grade A, B, or C hip (7). However, at present, there is no consensus on the best methodology for the early evaluation or conclusive assessment of the usefulness of various evaluable parameters (6, 8). Additionally, the evaluation of parameters is complicated by breed differences and the corresponding cutoff values that delineate a “healthy” hip joint from a “diseased” hip joint with increased laxity (7, 9, 10).

Kinetic motion analysis has become increasingly important in recent years to objectively describe the clinical effects of orthopedic diseases. Using this method, the ground reaction forces (GRF) are evaluated using force- or pressure-measurement plates. Numerous studies have used this non-invasive method to describe, for example, the compensatory effects of lameness (11–13) and therapeutic results (14–16). Additionally, the pressure distribution within the paws can be described by pedobarography with the help of pressure-measurement plates. Using this method, significant changes in the distribution of forces in the paws of orthopedically diseased dogs have been documented (17–20). Another more recently applied parameter is the center of pressure (COP), which is the location where the GRF vector acts on the ground (i.e., the application point of the force). The COP constantly changes its position to control the stability of the upright posture and gait pattern. This parameter can be evaluated when standing (statokinesiogram) and when in motion. Crucially, COP analysis reveals disturbances in postural control in human patients with hip dysplasia (HD) (21). Likewise, orthopedically diseased dogs show a significant increase in COP parameters, and COP parameter measurements are, therefore, considered useful in the evaluation of lameness (12, 18, 22). Furthermore, dogs with coxarthrosis show a significant increase in mediolateral COP deviation and COP-Area in the hindlimbs (12). While age-related changes in various COP parameters in horses have been documented between the first and the 5th months of life (23), few studies have evaluated the kinetic parameters of growing dogs. One study investigated whether the DI correlated with different kinetic variables, but no association was found, although this may reflect high data variability possibly linked to incompletely developed neuromuscular function and coordination of the puppies (24).

Based on the existing literature from both human and veterinary medicine, in this study, we aimed first time to obtain GRF and COP parameters for retriever dogs aged 4, 8, and 12 months. We hypothesized that even in clinically non-lame dogs, the COP parameter values of limbs with a CHD grade C or worse based on radiographic FCI hip scores would be increased compared to CHD grade A or B limbs. Results of the study could have potential of the early, non-invasive evaluation of future CHD risk in puppies and, thus, allowing early intervention.

## 2. Materials and methods

### 2.1. Dogs

The total number of participants included 51 puppies (Labrador Retrievers, *n* = 33; Golden Retrievers, *n* = 18) from the Austrian Retriever Club (ÖRC). For inclusion in the study, the breeding parents of a puppy had to have a breeding license with the ÖRC, and the owners had to have the intention to have an FCI hip and elbow radiograph as defined by the ÖRC, to be performed at the earliest age of 12 months. Puppies with disorders not related to the hip joint, such as osteochondrosis dissecans (OCD) of the shoulder joint and elbow dysplasia, were excluded from the study.

The dogs were presented at 4 (M1), 8 (M2), and 12 (M3) months of age. At each time point, the animals underwent clinical, orthopedic, and neurological examinations. Subsequently, the dogs underwent motion analysis on a Zebris pressure plate (FDM Type 2, Zebris Medical GmbH, Allgäu, Germany) with a measurement area of 203.2 × 54.2 cm containing 15,360 sensors with a sampling rate of 100 Hz. The plate was mounted in the middle of a 9-m runway and covered with a rubber mat (1 mm thickness) to hide the measurement area and prevent slipping. A camera (Panasonic NV-MX500) was used to obtain the measurements. The collected data were analyzed using custom software (Pressure Analyzer 4.3.3; Michael Schwanda).

### 2.2. Measurement procedure

The measurements were performed while walking and trotting. In each case, the dog and owner were allowed to familiarize themselves with the measuring room and explore it playfully. As soon as the dog felt comfortable, the measurement and data collection began. To ensure that the animal moved straight across the force plate with the head in a straight and forward position, an assistant stood directly at the opposite end of the force plate and encouraged the animal to walk or trot toward them while keeping eye contact with the dog. If free movement without a leash due to the individual behavior of the animal was not possible, the measurements were performed with a leash. To minimize the influence of leashes, dogs were trained to walk or trot in a smooth and harmonious gait pattern without pulling on the leash before the analysis began. At least five passes with valid steps over the pressure plate were performed during walking and trotting. Valid measurements were performed if the animal walked or trotted in a straight line with the head in a straight and forward position, without an apparent change in speed. In cases where the dog was led over the plate with a leash, the leash must have swung freely without pulling to be counted as a valid pass. Passes were judged as failed when the study participant did not move straight across the measurement plate (e.g., when it left the plate or entered it obliquely from the side) or held its head to the side, up, or down relative to the floor. Passes where the dog stopped, slowed down, sped up, or sat down, were also excluded. To be included, the speed at which a dog crossed the plate had to be within ± 0.3 m/s when walking, with a maximum of 0.5 m/s when trotting and with an acceleration of ± 0.5 m/s^2^ (25–27).

Radiological assessment was performed from the age of 12 months (M3), and this followed the FCI standard. Radiographic analysis was performed approximately 2 weeks before or after M3. The dog owners were allowed to have the examination performed at the veterinarian of their choice, but all of the resulting radiographs were sent to us for evaluation. Evaluation of the hip and elbow joints was performed by a certified radiologist (MG) according to the standard procedure of the FCI. The radiologist had no information about the results of the gait analysis. A symmetric ventrodorsal view of the coxofemoral joints and pelvis with caudally extended hind legs positioned parallel to the table (position I) was used for screening. Congruity of the joint space, shape and (abnormal) sclerosing of the cranial acetabular rim, shape, and density of the femoral head and neck, femoral head center in relation to the dorsal acetabular rim, radiographic signs of arthrosis, and, finally, the Norberg angle, were used to grade each hip joint as grade A (free of CHD), B (borderline), C (mild), D (moderate) to E (severe CHD). Furthermore, the presence or absence of a lumbosacral transitional vertebra was verified. Mediolateral and craniocaudal radiographs of both elbows were monitored for new bone formation, isolated anconeal processes, fractured medial coronoid processes, incongruity, osteochondrosis (dissecans), contact lesions, and incomplete humeral condyle ossification to obtain a final grade ranging from ED 0 (free of elbow dysplasia) to ED III (grade 3, severe elbow dysplasia).

### 2.3. Data evaluation

Through video analysis, the footprints displayed on the custom Pressure Analyzer software (version 4.3.3, Michael Schwanda) were manually assigned to the corresponding hindlimb. For data analysis, the limbs were divided into two groups—those that were evaluated at the FCI examination with HD grade A or B (sound limb) and those with a HD grade of C or worse (diseased limb).

Subsequently, the following parameters were obtained (12, 28, 29):

- Speed (m/s) and acceleration (m/s^2^) for the left hindlimb.
- Peak vertical force (PFz, N) and vertical impulse (IFz, Ns) for each hind limb normalized to the total force exerted by the hindlimbs (PFz %, IFz %), using the following formula:

$$\begin{array}{l} Value\;in\;\%\;of\;total\;force\;=\;\left(\frac{XFz\text{\_}LR\;or\;LL}{XFz\text{\_}LR+XFz\text{\_}LL}\right)\times 100 \end{array}$$

- where XFz = mean value of PFz or IFz of the valid steps, LR = right hindlimb, and LL = left hindlimb.
- Stance phase duration (SPD): The mean duration of the stance phase(s) of each limb was normalized to the total duration of the stance phase (SPD %) following the previous formula.
- Time of occurrence of PFz (TPFz) as a percentage (%) of the stance phase of the respective limb.
- Paw contact area (cm^2^): The area of force application.
- Center of pressure area (COP-Area): The surface that includes all points of the COP normalized to the paw contact area and expressed as a percentage (%).
- Mediolateral (COP-Med-lat) and craniocaudal COP (COP-Cran-caud) displacements: The difference between the maximum positive and negative excursions along the craniocaudal and mediolateral axes, respectively, expressed as a percentage (%) of the maximum width or length of the paw contact area.
- COP-Radius (mm): The mean of the distance of all COP points to the center point of all COP points, normalized to the paw contact area and expressed as a percentage (%).
- COP-Speed: The mean speed (distance/time) of the movement of the COP (mm/s).

### 2.4. Statistical analysis

All statistical analyses were performed using IBM SPSS v28. For every single parameter, the difference between hips (sound with FCI scoring A and B and diseased with FCI scoring C and worse), speed (walk and trot), and time (4, 8, and 12 months) were analyzed separately for each of the two breeds using a linear mixed effect model. Specific *post-hoc* comparisons were performed using Sidak's alpha correction procedure. The assumption of normal distribution was assessed using the Shapiro–Wilk test. A *p*-value of < 5% (*p* < 0.05) was considered statistically significant.

## 3. Results

The demographic data of the dogs at the time of measurement are shown in Table 1. Based on the inclusion criteria, one Labrador Retriever in the 8th month of life had to be excluded because of OCD in one shoulder, and one Golden Retriever was excluded because he displayed elbow dysplasia grade 2, as detected by the FCI investigation. Therefore, data were evaluated based on a total of 49 dogs [98 hip joints from 32 Labrador (17 female, 15 male) and 17 Golden Retrievers (10 females, 7 males).

**Table 1:** Demographic data of the studied dogs at three measurement points (M1 = 4 months, M2 = 8 months, and M3 = 12 months of age).

	**Labrador Retriever (*****n*** = **32)**			**Golden Retriever (*****n*** = **17)**		

**Evaluation**	**M1**	**M2**	**M3**	**M1**	**M2**	**M3**
Body mass (kg ± SD)	13.34 ± 3.17	23.62 ± 4.23	26.35 ± 4.80	16.54 ± 3.84	27.64 ± 5.22	29.07 ± 5.32
Mean age (weeks ± SD)	18.41 ± 2.17	34.69 ± 1.00	59.09 ± 9.30	19.00 ± 1.80	34.82 ± 1.19	60.76 ± 11.32

### 3.1. Linear mixed effect model

The linear mixed effect model showed that except for COP-Med-lat (*p* = 0.065), the healthy or diseased status of the dogs had a significant effect on all the COP parameters for the entire population (*p* < 0.001). Regarding the GRF parameters, health status had a significant effect on IFz (*p* = 0.040) and TPFz (*p* = 0.028). There were also significant effects of breed on COP-Cran-caud, COP-Radius, and COP-Speed (*p* < 0.001). Speed of gait (at walk and trot) had a significant influence on all the COP parameters and TPFz (*p* < 0.001). The time of measurement (4, 8, and 12 months) also had a significant influence on all of the COP parameters (*p* < 0.001) but not GRF. Breed influenced three out of the five COP parameters (COP-Cran-caud, COP-Radius, and COP-Speed; *p* < 0.001) and TPFz (*p* = 0.046). Thus, subsequent evaluations were performed separately for the two breeds.

### 3.2. Labrador Retrievers

#### 3.2.1. Clinical, orthopedic, and neurological examination

Clinical, orthopedic, and neurological examinations of the 32 Labradors did not reveal any abnormalities. As previously noted, one dog was excluded because of an OCD at M2.

#### 3.2.2. Hip scoring

FCI examination of the 64 hip joints revealed the following results: 31 were rated as grade A, 16 as B, 12 as C, 2 as D, and 3 as E. Therefore, 47 hip joints were scored as sound and 17 as diseased. Among the 17 diseased hips 5 animals had unilateral and 6 bilateral CHD. Eight dogs displayed a lumbosacral transitional vertebra, 5 in dogs with sound hip joints (3 type 1 and 2 type 3) and 3 dogs with bilateral FCI scoring C (all type 1).

#### 3.2.3. Speed and acceleration

The speed and acceleration data are presented in Table 2. All values were within the acceptable ranges described in Section 2.2.

**Table 2:** Speed (v) and acceleration (a) (mean ± SD) (calculated for the left hind limb) when walking and trotting for Labrador Retrievers (*n* = 32) at the three measurement points (M1 = 4 months, M2 = 8 months, and M3 = 12 months of age).

**Measurement**		**M1**	**M2**	**M3**
Walk	a (m/s^2^)	0.00 ± 0.02	−0.01 ± 0.04	0.01 ± 0.03
	v (m/s)	1.03 ± 0.09	1.17 ± 0.10	1.23 ± 0.12
Trot	a (m/s^2^)	0.01 ± 0.08	0.01 ± 0.12	0.07 ± 0.20
	v (m/s)	2.01 ± 0.17	2.28 ± 0.18	2.41 ± 0.26

v = speed (m/s) and a = acceleration (m/s^2^) for the left hindlimb.

#### 3.2.4. Differences between sound and diseased limbs

With regard to GRF and SPD, no differences were found in any of the measurements between limbs classified as sound and diseased. The only exception was TPFz, which was reached earlier by the sound limbs at M3 during walking (*p* = 0.023) and later in trot (*p* = 0.051) (Table 3).

**Table 3:** Descriptive statistics (mean ± SD) for walking and trotting Labrador Retrievers (*n* = 32) at three measurement points (M1 = 4 months, M2 = 8 months, and M3 = 12 months of age) in sound and diseased limb groups.

**Measurement**		**M1**		**M2**		**M3**	

**Gait**	**Parameter**	**Sound**	**Diseased**	**Sound**	**Diseased**	**Sound**	**Diseased**
Walk	COP-Area (%)	1.84 ± 0.44^*^^#^^¥^	2.24 ± 0.66^*^^#^^¥^	0.97 ± 0.27^*^^#^	1.20 ± 0.37^*^^#^	0.84 ± 0.23^(*)^^¥^	0.98 ± 0.33^(*)^^¥^
	COP-Cran-caud (%)	35.23 ± 5.17^*^^#^^¥^	38.32 ± 6.87^*^^#^^¥^	23.26 ± 3.36^*^^#^	25.99 ± 4.52^*^^#^	21.87 ± 3.26^*^^¥^	24.76 ± 3.56^*^^¥^
	COP-Med-lat (%)	8.19 ± 1.82^#^^¥^	8.60 ± 1.65^#^^¥^	5.80 ± 1.20^*^^#^	6.98 ± 1.00^*^^#^	5.44 ± 1.37^¥^	6.11 ± 2.29^¥^
	COP-Radius (%)	0.22 ± 0.03^*^^#^^¥^	0.25 ± 0.07^*^^#^^¥^	0.13 ± 0.02^*^^#^	0.15 ± 0.03^*^^#^	0.12 ± 0.02^¥^	0.13 ± 0.02^¥^
	COP-Speed (mm/s)	96.92 ± 10.07^*^^#^^¥^	109.24 ± 21.63^*^^#^^¥^	68.84 ± 8.72^*^^#^	78.99 ± 15.00^*^^#^	68.08 ± 10.39^*^^¥^	76.16 ± 9.65^*^^¥^
	IFz (%TF)	50.00 ± 1.32	49.99 ± 1.46	49.98 ± 0.88	50.04 ± 0.63	50.06 ± 0.98	49.83 ± 1.51
	PFz (%TF)	50.05 ± 1.47	49.85 ± 1.17	49.98 ± 1.39	50.07 ± 1.15	50.08 ± 1.36	49.77 ± 1.85
	SPD (%)	49.99 ± 0.71	50.03 ± 0.79	50.00 ± 0.57	50.00 ± 0.38	49.99 ± 0.52	50.03 ± 0.78
	TPFz (%SPD)	22.01 ± 2.43	22.62 ± 3.47	23.99 ± 4.68	23.70 ± 3.08	23.73 ± 2.96^*^	25.37 ± 3.39^*^
Trot	COP-Area (%)	0.70 ± 0.30	0.80 ± 0.24	0.75 ± 0.34	0.85 ± 0.30	0.64 ± 0.34^*^	0.92 ± 0.34^*^
	COP-Cran-caud (%)	25.46 ± 4.48^*^^#^^¥^	28.22 ± 4.63^*^^#^^¥^	21.62 ± 3.77^#^^£^	22.76 ± 3.90^#^	18.86 ± 5.39^*^^¥^^£^	22.68 ± 5.08^*^^¥^
	COP-Med-lat (%)	4.94 ± 1.47	5.65 ± 1.85	5.27 ± 1.44	5.74 ± 1.41	5.93 ± 2.48	6.31 ± 1.98
	COP-Radius (%)	0.15 ± 0.02^(*)^^#^^¥^	0.16 ± 0.03^(*)^^#^^¥^	0.12 ± 0.02^#^^£^	0.12 ± 0.02^#^	0.11 ± 0.02^*^^¥^^£^	0.12 ± 0.02^*^^¥^
	COP-Speed (mm/s)	238.60 ± 32.52^#^^¥^	244.48 ± 22.42^#^^¥^	178.94 ± 29.26^#^	186.47 ± 22.70^#^	171.64 ± 30.14^¥^	183.92 ± 26.08^¥^
	IFz (%TF)	50.08 ± 1.20	49.79 ± 1.78	50.17 ± 1.89	49.52 ± 1.89	50.27 ± 1.85	49.24 ± 2.25
	PFz (%TF)	50.07 ± 0.87	49.81 ± 1.30	50.07 ± 1.23	49.80 ± 1.63	50.17 ± 1.50	49.53 ± 1.34
	SPD (%)	50.00 ± 0.89	50.00 ± 1.18	50.07 ± 1.18	49.81 ± 0.51	50.02 ± 0.85	49.94 ± 0.80
	TPFz (%SPD)	41.51 ± 1.85^#^^¥^	42.00 ± 2.10^¥^	41.85 ± 1.85^#^	40.61 ± 2.02	42.50 ± 2.27^*^^¥^	41.34 ± 2.11^*^^¥^

* Significant difference between groups (*p* < 0.05),

(*) *p* = 0.06;

# significant difference between M1 and M2,

¥ significant difference between M1 and M3,

£ significant difference between M2 and M3.

**Figure 1:**
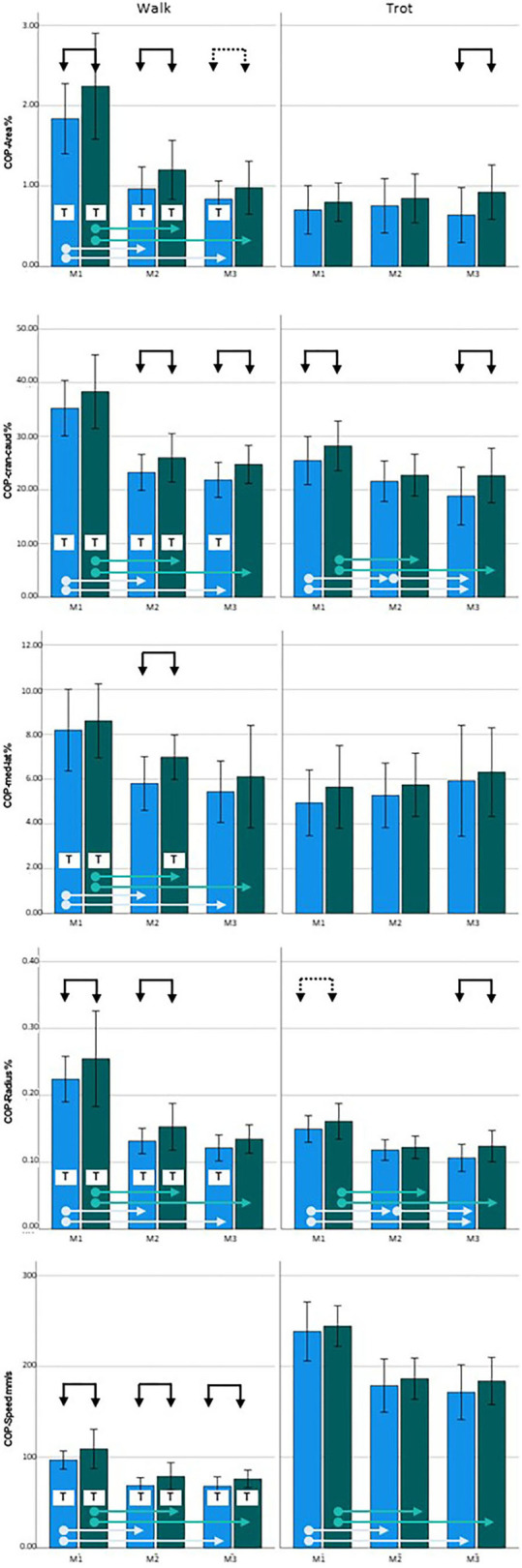
Center of pressure (COP) data for Labrador Retrievers: **Left** = walking, **right** = trotting; **blue** = sound limbs, **green** = diseased limbs; M1 = 4 months; M2 = 8 months; M3 = 12 months. Black arrows indicate significant differences between sound and diseased limb groups; dotted black arrows indicate *p* = 0.06; light blue and green arrows indicate significant differences between measurement points in the sound and diseased limb groups, respectively. T = significant difference between walking and trotting.

At M1, COP values were increased for three of the five parameters in the diseased legs (COP-Area, *p* = 0.033; COP-Radius, *p* = 0.023; and COP-Speed, *p* = 0.004) in walking and one of the five parameters in trot (COP-Cran-caud, *p* = 0.022; COP-Radius was just short of significance, *p* = 0.06). At M2, all of the parameters showed increased values during walking (COP-Area, *p* = 0.016; COP-Cran-caud, *p* = 0.004; COP-Med-lat, *p* = 0.002; COP-Radius, *p* = 0.001; COP-Speed, *p* < 0.001), but no differences were detected In trot. At M3, significant differences between limbs were identified for two parameters during walking (COP-Cran-caud, *p* = 0.002; COP-Speed, *p* = 0.006; COP-Area was just short of significance, *p* = 0.064) and three parameters when trotting (COP-Area, *p* < 0.001; COP-Cran-caud, *p* = 0.002; COP-Radius, *p* = 0.002; Table 3; Figure 1).

#### 3.2.5. Development over time

For both sound and diseased limbs, there were no significant differences in PFz, IFz, and SPD between the measurement periods. TPFz occurred significantly later during walking in M2 (*p* = 0.023) and M3 (*p* = 0.010) than in M1 in the sound limbs, and between M1 and M3 in the diseased limbs (*p* = 0.008) (Table 3).

Considering the development of the COP over time during walking, all of the five COP parameters had significantly higher values at M1 than at M2 and M3 (both limb groups, *p* < 0.001), while there were no differences between M2 and M3. When trotting, COP-Area and COP-Med-lat showed stable values over time, while the sound limb group showed significantly higher values for the other three parameters at M1 than at M2 and M3 (*p* < 0.001). In contrast, COP-Cran-caud (*p* = 0.006) and COP-Radius (*p* = 0.008) decreased between M2 and M3. While the diseased legs showed similar overall trends, the decrease in COP-Cran-caud and COP-Radius values between M2 and M3 was not observed (*p* < 0.000–0.004) (Table 3, Figure 1).

#### 3.2.6. Comparison of walk and trot

In both the sound and diseased limb groups, PFz, IFz, and SPD did not show significant differences between walking and trotting, but TPFz occurred significantly later during trotting in both limb types (*p* < 0.001) (Table 3).

Almost all of the COP values of the sound legs were significantly higher when walking than when trotting at each of the measurement points (COP-Area, *p* < 0.001, *p* = 0.005, and *p* = 0.001; COP-Cran-caud, *p* = < 0.001, *p* = 0.036, and *p* = 0.001; and COP-Radius *p* < 0.001, *p* = 0.001, and *p* = 0.008 at M1, M2, and M3, respectively). COP-Med-lat was higher during walking only at M1 (*p* < 0.001). COP-Speed, was lower during walking than during trotting (all three points, *p* < 0. 001). In the diseased limb groups, COP-Speed was lower during walking than during trotting at each measurement period (*p* < 0.001). The values of all the other COP parameters were higher during walking than when trotting at M1 and M2 (COP-Area *p* < 0.001 and *p* = 0.005; COP-Cran-caud *p* < 0.001 and *p* = 0.015; COP-Med-lat *p* < 0.001 and *p* = 0.023; and COP-Radius *p* < 0.001 and *p* < 0.001, respectively) but not at M3 (Table 3, Figure 1).

### 3.3. Golden Retrievers

#### 3.3.1. Clinical, orthopedic, and neurological examination

The clinical, orthopedic, and neurological examinations of the 17 Golden Retrievers did not reveal any abnormalities. However, as previously noted, one dog was excluded because of elbow dysplasia seen in radiographs at M3.

#### 3.3.2. Hip scoring

The FCI examination of the 34 hip joints revealed the following results: 11 were rated as grade A, 11 as B, 8 as C, and 4 as D. Therefore, 22 hip joints were scored as sound and 12 as diseased. Among the 12 diseased hips 4 animals had unilateral and 4 bilateral CHD. No lumbosacral transitional vertebras were detected.

#### 3.3.3. Speed and acceleration

The speed and acceleration data are presented in Table 4. All values were within the acceptable ranges described in Section 2.2.

**Table 4:** Speed (v) and acceleration (a) (mean ± SD) (calculated for the left hind limb) when walking and trotting for Golden Retrievers (*n* = 17) at the three measurement points (M1 = 4 months, M2 = 8 months, and M3 = 12 months of age).

**Measurement**		**M1**	**M2**	**M3**
Walk	a (m/s^2^)	0.00 ± 0.03	0.01 ± 0.02	0.01 ± 0.02
	v (m/s)	1.00 ± 0.10	1.10 ± 0.12	1.17 ± 0.10
Trot	a (m/s^2^)	−0.03 ± 0.11	0.04 ± 0.12	0.00 ± 0.08
	v (m/s)	1.96 ± 0.22	2.18 ± 0.17	2.18 ± 0.23

v = speed (m/s) and a = acceleration (m/s^2^) for the left hindlimb.

#### 3.3.4. Differences between sound and diseased limbs

None of the GRF parameters showed significant differences between diseased and sound legs (Table 5) except for TPFz, which was significant later at M3 in the sound limbs when trotting (*p* = 0.009).

**Table 5:** Descriptive statistics (mean ± SD) for walking and trotting Golden Retrievers (*n* = 17) at three measurement points (M1 = 4 months, M2 = 8 months, and M3 = 12 months of age) in sound and diseased limb groups.

**Measurement**		**M1**		**M2**		**M3**	
**Gait**	**Parameter**	**Sound**	**Diseased**	**Sound**	**Diseased**	**Sound**	**Diseased**
Walk	COP-Area (%)	1.62 ± 0.75^*^^#^^¥^	2.47 ± 1.09^*^^#^^¥^	0.89 ± 0.42^*^^#^	1.38 ± 0.52^*^^#^	0.82 ± 0.38^*^^¥^	1.14 ± 0.31^*^^¥^
	COP-Cran-caud (%)	36.15 ± 6.46^*^^#^^¥^	43.55 ± 7.50^*^^#^^¥^	24.76 ± 4.06^*^^#^	30.80 ± 4.63^*^^#^	23.35 ± 3.95^*^^¥^	29.47 ± 4.54^*^^¥^
	COP-Med-lat (%)	9.02 ± 1.92^#^^¥^	8.87 ± 1.74^¥^	6.36 ± 1.74^*^^#^	7.50 ± 2.11^*^	5.85 ± 2.24^¥^	6.40 ± 1.58^¥^
	COP-Radius (%)	0.22 ± 0.05^#^^¥^	0.24 ± 0.04^#^^¥^	0.14 ± 0.02^*^^#^	0.17 ± 0.03^*^^#^	0.15 ± 0.06^¥^	0.15 ± 0.02^¥^
	COP-Speed (mm/s)	102.17 ± 16.40^*^^#^^¥^	114.24 ± 14.87^*^^#^^¥^	73.53 ± 11.73^*^^#^	86.44 ± 10.01^*^^#^	73.59 ± 12.20^*^^¥^	86.96 ± 9.75^*^^¥^
	IFz (%TF)	49.88 ± 0.96	50.22 ± 1.43	49.98 ± 0.83	50.05 ± 0.61	50.15 ± 1.29	49.72 ± 0.92
	PFz (%TF)	49.83 ± 1.53	50.32 ± 2.35	49.95 ± 0.76	50.10 ± 1.25	50.11 ± 1.15	49.80 ± 0.96
	SPD (%)	49.96 ± 0.78	50.08 ± 0.38	49.98 ± 0.43	50.03 ± 0.43	50.04 ± 0.64	49.92 ± 0.76
	TPFz (%SPD)	24.23 ± 2.17	24.21 ± 4.11	25.80 ± 2.67	23.26 ± 2.24	26.40 ± 2.31	24.83 ± 1.68
Trot	COP-Area (%)	0.88 ± 0.48	0.95 ± 0.33	0.89 ± 0.47	1.01 ± 0.43	0.73 ± 0.34	0.84 ± 0.28
	COP-Cran-caud (%)	28.06 ± 3.95^*^^¥^	32.68 ± 4.31^*^^#^^¥^	25.53 ± 4.15^*^	28.50 ± 4.53^*^^#^	22.54 ± 3.68^*^^¥^	26.85 ± 4.15^*^^¥^
	COP-Med-lat (%)	5.95 ± 2.94	5.25 ± 1.61	5.62 ± 2.11	5.43 ± 2.40	5.38 ± 2.07	4.61 ± 0.95
	COP-Radius (%)	0.16 ± 0.02^#^^¥^	0.17 ± 0.02^#^^¥^	0.14 ± 0.02^#^	0.14 ± 0.02^#^	0.13 ± 0.02^¥^	0.13 ± 0.02^¥^
	COP-Speed (mm/s)	251.94 ± 38.41^*^^#^^¥^	286.94 ± 40.21^*^^#^^¥^	182.16 ± 27.89^*^^#^	210.41 ± 54.31^*^^#^	172.97 ± 22.53^*^^¥^	200.15 ± 37.11^*^^¥^
	IFz (%TF)	50.16 ± 1.03	49.71 ± 0.96	50.19 ± 1.82	49.65 ± 1.29	50.08 ± 2.04	49.85 ± 3.40
	PFz (%TF)	50.02 ± 0.76	49.97 ± 0.75	50.00 ± 1.29	49.99 ± 0.97	49.92 ± 1.23	50.15 ± 3.09
	SPD (%)	50.12 ± 0.78	49.77 ± 0.84	50.12 ± 1.38	49.78 ± 1.18	50.14 ± 0.90	49.75 ± 2.70
	TPFz (%SPD)	40.71 ± 1.78	40.66 ± 1.95^#^^¥^	39.45 ± 4.18	37.53 ± 5.83^#^	39.86 ± 1.79^*^	37.87 ± 1.51^*^^¥^

* Significant difference between groups (*p* < 0.05),

# significant difference between M1 and M2,

¥ significant difference between M1 and M3.

**Figure 2:**
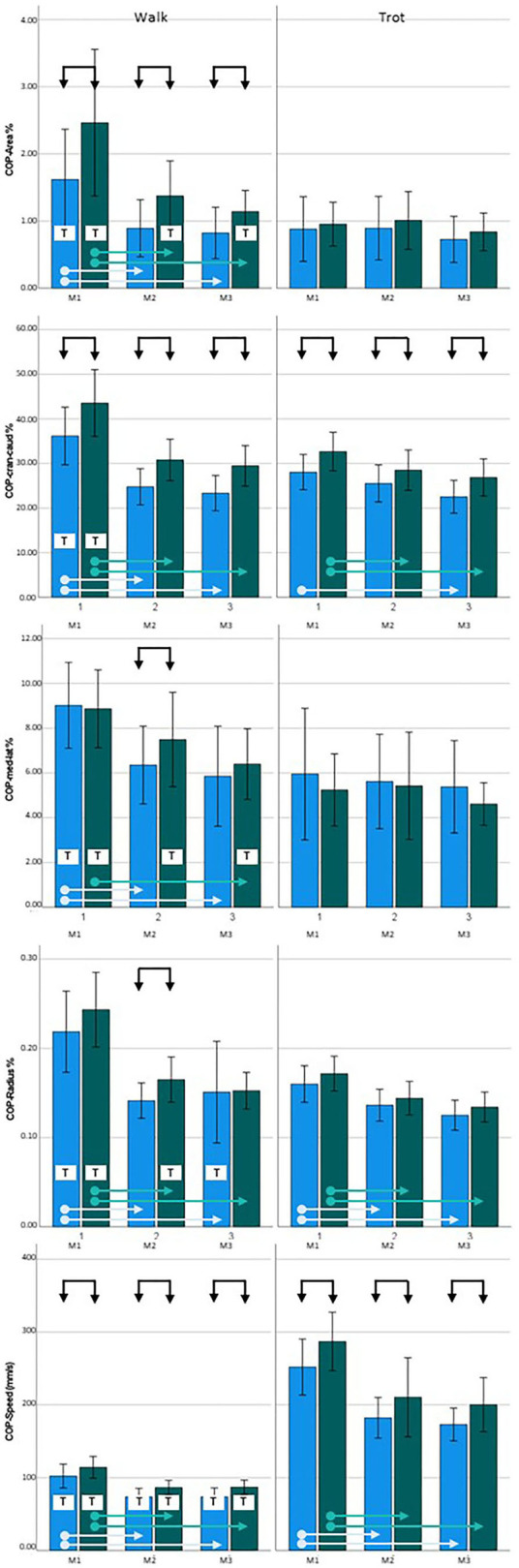
Center of pressure (COP) data for Golden Retrievers. **Left** = walking, **right** = trotting; **blue** = sound limbs, **green** = diseased limbs; M1 = 4 months; M2 = 8 months; M3 = 12 months. Black arrows indicate significant differences between sound and diseased limb groups; dotted black arrows indicate *p* = 0.06; light blue and green arrows indicate significant differences between measurement points in the sound and diseased limb groups, respectively. T = significant difference between walking and trotting.

At M1, three of the COP parameters showed significantly higher values in the diseased limb group when walking (COP-Area, *p* < 0.001; COP-Cran-caud, *p* = 0.001; COP-Speed, *p* = 0.023). When trotting, higher values were also obtained for COP-Cran-caud (*p* = 0.004) and COP-Speed (*p* = 0.004). At M2, all of the COP parameters showed higher values in the diseased limb group during walking (COP-Area, *p* < 0.001; COP-Cran-caud, *p* < 0.001; COP-Med-lat, *p* = 0.020; COP-Radius, *p* = 0.005; COP-Speed, *p* < 0.001) and two parameters had higher values when trotting (COP-Cran-caud, *p* = 0.035; COP-Speed, *p* = 0.020). At M3, three of the COP parameters had higher values when walking (COP-Aare, *p* = 0.003; COP-Cran-caud, *p* < 0.001; COP-Speed, *p* = 0.001) and two had higher values when trotting (COP-Cran-caud, *p* = 0.011; COP-Speed, *p* = 0.007; Table 5; Figure 2).

#### 3.3.5. Development over time

In both the sound and diseased limb groups, PFz, IFz, and SPD did not significantly differ between the measurement points. In the diseased limb group, TPFz occurred significantly earlier when trotting at M2 (*p* = 0.011) and M3 (*p* = 0.002) than at M1 (Table 5).

During walking, all of the COP parameters for the sound limb group had significantly higher values at M1 than at M2 and M3 (*p* < 0.001) but remained constant between M2 and M3. Similar results were observed in the diseased limb group (*p* < 0.001), except the COP-Med-lat was only significantly different between the M1 and M3 measurements (*p* = 0.003). When trotting, COP-Area and COP-Med-lat did not change over time in either the sound or diseased limb group. In the sound limb group, COP-Radius and COP-Speed values at M1 were higher than those at M2 and M3 (*p* < 0.001), and COP-Cran-caud differed only when comparing M1 to M3 (*p* < 0.001). In the diseased limb group, the three of the COP parameters were consistently higher at M1 than at M2 and M3 (COP-Cran-caud, *p* = 0.034 and *p* = 0.005; COP-Radius, *p* = 0.001 and < 0.001; COP-Speed, *p* < 0.001 and *p* < 0.001, respectively; Table 5; Figure 2).

#### 3.3.6. Comparison of walk and trot

For both the sound and diseased limb groups, PFz, iFz, and SPD did not significantly differ between walking and trotting, while TPFz occurred significantly later during trotting in both groups (*p* < 0.001) (Table 5).

COP-Speed values when walking were significantly lower than when trotting for both limb groups and at all three measurement points (*p* < 0.001). All of the other measured parameters showed significantly higher values for both limb groups at M1 when walking compared to trotting (*p* < 0.001). At M2, there were no differences for cop-Area; COP-Cran-caud, COP-Med-lat and COP-Radius in the sound limb group, while in the diseased limb group, the COP-Area (*p* = 0.015), COP-Med-lat (*p* = 0.002), and COP-Radius (*p* = 0.010) values were higher during walking. At M3, the sound limb group had higher COP-Radius values when walking compared to trotting (*p* = 0.002), and in the diseased limb group, the COP-Area (*p* = 0.013) and COP-Med-lat (*p* = 0.029) values were significantly higher when walking (Table 5, Figure 2).

### 3.4. Synthesis

At the age of 8 months (M2), in both breeds, all of the examined COP-Parameters had significantly higher values for the diseased limb group during walking. At 4 (M1) and 12 (M3) months of age, this difference was less pronounced, but three of the five measured parameters were still affected when walking. As COP-Area and COP-Speed were affected at all three measurement points when walking, these represent the most reliable parameters for early detection of CHD. In contrast, we found few differences in COP parameters between the sound and diseased limb groups of Labrador Retrievers when trotting: at 4 months, COP-Cran-caud values were different (and COP-Radius was just short of significance); at 8 months, none of the parameters were different; and at 12 months, three parameters (COP-Area, COP-Cran-caud, and COP-Radius) were affected. In the Golden Retrievers, the COP-Radius values when walking and trotting, and the COP-Speed when trotting, were consistently higher in the diseased limb group at all measurement points.

## 4. Discussion

We evaluated the COP and GRF parameters of Labrador and Golden Retrievers at 4, 8, and 12 months of age using a pressure plate. We hypothesized that even in non-lame dogs, the COP parameter values of limbs assessed with a HD grade of C or worse (i.e., diseased according to the FCI radiographic standards) would be higher than those of HD grade A or B (i.e., sound). Our results confirm this hypothesis.

The differences in COP parameters between limbs with sound and diseased hip joints can be interpreted with respect to possible biomechanical adaptations (18, 22) as well as being an indicator of reduced stability (18, 21). Comparisons with the existing literature are difficult because veterinary studies have typically used only adult lame animals when examining COP. Additionally, in some studies (18, 22), measurements were performed while the animal was standing and not walking/trotting, with three measurements of 20 s each performed when the dogs were not permitted to move. Because this is very difficult to achieve with puppies, we made our measurements while the dogs were in motion.

Nevertheless, the changes in outcome parameters presented in our study can be related to the existing literature. In particular, the findings of Poy et al. (30) provide insight into why differences in COP parameters and TPFz were observed. Dogs with CHD show greater adduction, a greater range of motion in abduction-adduction, and greater lateral movement of the pelvis. CHD leads to complex changes in the kinematics of the affected joint and the other joints of the limb. Additionally, changes in angularities and angular velocities in diseased individuals have been described (31–34). Those kinematic changes explain the differences in COP-Area and mediolateral COP displacement, as already described for lame dogs (12). The increased abduction-adduction seems to cause a greater excursion of the COP in mediolateral direction (and thus also of the COP-Area). It is noteworthy that these changes (especially of the COP-Area) also occurred without a lameness in the animals of our study. Here, it is additionally striking that we could also present increases in COP-Cran-caud, which certainly also contributed to the increased COP-Area, but probably also to the COP-Radius. However, it is unclear why this change in COP-Cran-caud was not also presented in lame dogs (12). Several considerations can be used as a basis for discussion. Dysplastic dogs show increased extension of the hip joint at the end of the stance phase with a concomitant faster extension during the stance phase. The stifle joint is more flexed during and flexed faster at the end of the stance phase. The tarsal joint is more flexed at the beginning of the stance phase and its extension at the end of the stance phase is slowed down. This compensatory interaction of the joints may lead to the fact that in lame dogs the craniocaudal excursion of the COP is not altered. In the young animals used in this study, it could be assumed that the lameness-free gait caused kinematic changes that led to increases in COP-Cran-Caud. This conclusion could be supported by the significant difference in TPFz, which was only observed at M3. Possibly, this change indicates increased adaptations of the gait pattern, especially since this has not been described before (34). The relationship between kinematics and COP is also supported by Lopez et al. (22). These authors interpreted the altered COP values of dogs with elbow joint disease as a result of modified kinematics, especially in the case of the larger caudal margin (i.e., the distance between the most caudal limit of the paw print and the most caudal limit of the limb COP path); notably, we did not measure this parameter in our study. To prove all these assumptions, kinematic studies with growing dogs should be performed in the future.

Notably, in one study, postural changes in human patients with osteoarthritis of the hip joints due to pathologies of the joint structures impair proprioception and postural stability (21), although patients were measured in the standing position, which limits kinematic changes in the motion sequence. Thus, our results may reflect a combination of the hip joint not developing normally (and, correspondingly, disturbed proprioceptive input) and altered kinematics. Further studies are now needed to address this. Muscling of the affected limb may also have influenced our results, which increases in the growing animal alongside possible muscular adaptions. This was supported by evidence that the longissimus dorsi muscle shows increased activity contralateral to the affected limb, and that lame animals rotate their limbs mediolaterally (35). To address this further, the extent to which alterations in muscle activity are observed in lameness-free growing dogs should be investigated.

In addition to the significant differences between the diseased and sound limb groups, we found striking differences in the COP parameter values between the measurement time points. Especially when walking, the COP values were significantly lower from M2 (8 months of age). This likely indicates increased stability of the gait pattern of developing puppies, with similar observations reported for horses; newborn foals show a relative increase in craniocaudal COP, which decreases rapidly in the first week of life and slowly stabilizes over the following months (23). In our dogs, this phenomenon also occurred when trotting, but not for all of the measured parameters. It may be that the two-beat trot is easier for animals to control than the more complicated footing pattern of walking. Indeed, this was supported by the fact that the COP values were higher during walking (with the logical exception of COP-Speed) than during trotting. However, further studies are needed to investigate the extent to which the shape of the paws also changes during growth, as this may influence COP.

As with previous studies, our study demonstrates that the homogeneity of the cohort group is a critical factor (36, 37). Specifically, the breed had a statistically significant effect on some of the measured parameters, although the results may have been influenced by different group sizes. To date, no studies have been dedicated to comparing the COP in different breeds, which may be an interesting area of future research.

While our results indicate that even in very young dogs, limbs later classified as CHD show detectable changes in COP parameters, several limitations should be acknowledged. First, the number of dogs examined was relatively small, and more limbs were judged to be healthy than diseased, meaning that different degrees of CHD could not be examined separately. Although grouping hips rated C or worse is not uncommon (6), it is unfortunate that we could not determine the extent to which hips assessed as HD grades D and E contributed to the results. Further studies should therefore seek to obtain data from more animals to further build on our results.

Indeed, compensatory mechanisms may occur between limbs that influence COP measurements, and even in studies with larger sample sizes, interpretations will be complicated by bilateral disease. In our study, 50% of affected animals had bilateral disease and 50% had unilateral disease. The interpretation of our results is further complicated by the inability to examine the diseased and sound contralateral sides of the same animals. It can be assumed that bilateral disease leads to compensatory mechanisms that are different from those in animals with unilateral disease. To verify this in further studies animals with unilateral and bilateral disease should be compared. Furthermore, the presence of transitional vertebrae in the Labrador Retrievers may have influenced the COP parameters. However, more transitional vertebrae occurred in the dogs with healthy hip joints. In any case, this issue should be addressed in further studies and COP measurements should be performed in dogs with different types of transitional vertebrae and healthy dogs.

Additionally, the rather small differences (despite statistically significant differences) in the COP parameters of the healthy and diseased hip joints should be considered. This makes it difficult to establish cut-off values that allow a tentative diagnosis. It should be considered whether further clinical examination methods, such as the flexion test (38), could be used to allow a clearer distinction between healthy and diseased joints.

One way to achieve deeper insight into the biomechanics of dogs would be to implement early diagnostic radiological procedures, as the evaluation of the distraction index (4). This could yield correlations between measured COP values and the current status of the hip joint. And, if studies with more animals confirm our results, COP measurements together with early radiological diagnostics the distraction index could further improve the prediction of CHD.

Another possibility for early diagnosis is ultrasonography, which can be performed without sedation. However, this method is not recommended due to a lack of correlation of the measurement results with the final CHD grade in the adult dog (39, 40). However, it should be investigated whether a combination of COP measurements and ultrasound would provide further indicators that could contribute to early diagnosis.

Overall, we suggest that COP measurements could be used to obtain useful indicators of potential CHD development in very young dogs. This could guide further early diagnosis based on radiological examination.

## Data availability statement

The original contributions presented in the study are included in the article/supplementary material, further inquiries can be directed to the corresponding author.

## Ethics statement

The animal study was reviewed and approved by Ethics and Animal Welfare Committee. Written informed consent was obtained from the owners for the participation of their animals in this study.

## Author contributions

BB and YV contributed to the study's conception and design. YV, BB, and CL performed measurements and evaluated the data. AT performed statistical analyses. MG evaluated and graded the radiographs. YV wrote the draft of the manuscript. MG, AT, CL, and BB wrote the manuscript. All authors contributed to manuscript revision and read and approved the submitted version.
